# Massive DVT from the proximal IVC to the pedal vein: Our approach using aspiration mechanical thrombectomy and open surgery thrombectomy

**DOI:** 10.1016/j.radcr.2023.02.021

**Published:** 2023-03-05

**Authors:** Jacub Pandelaki, Hariadi Hadibrata, Ivan Sini, Rajesh Kalwani, Prijo Sidipratomo, Heltara Ramandika, Dieby Adrisyel, Febian Sandra, Jason Jason

**Affiliations:** a Department of Radiology, Dr Cipto Mangunkusumo National General Hospital—Faculty of Medicine, Universitas Indonesia, Jl. Pangeran Diponegoro No.71, Kenari, Senen, Central Jakarta, Jakarta, Indonesia; b Bunda General Hospital, Jakarta, Indonesia; c Clerkship Program, Faculty of Medicine, Universitas Indonesia, Jakarta, Indonesia

**Keywords:** Deep vein thrombosis, Aspiration mechanical thrombectomy, Open surgical thrombectomy, Stent, Percutaneous transluminal angioplasty, Kissing catheter technique

## Abstract

Deep venous thrombosis might present in an acute condition requiring early thrombus removal. Several endovascular and surgical approaches are available with a short treatment time and minimal complications compared to pharmacotherapies. However, due to a lack of evidence, these are not the first treatment choice for deep vein thrombosis. Our case report showed a successful multimodality treatment for an acute-on-chronic massive deep vein thrombosis from the inferior vena cava to the pedal vein. A 47-year-old with chief complaints of cold, significant swelling, and severe pain in her left leg was diagnosed with deep vein thrombosis through Doppler ultrasound and contrast-enhanced computed tomography. The patient received aspiration mechanical thrombectomy with the “kissing catheter” technique, adjunctive stent, percutaneous transluminal angioplasty, and open surgical thrombectomy by Fogarty catheter without recurrence and complication.

## Introduction

Deep venous thrombosis (DVT) is a life-threatening condition with a significant mortality rate that accounts for more than 100,000 deaths and more than 250,000 hospital admissions annually in the United States. DVT is the formation of blood clots in the deep veins of the extremities; usually, the lower extremities [1]. DVT could cause an acute condition with symptoms of pain, swelling, erythema, or presents with systemic complications [2,3]. Although current standard practices revolve around pharmacotherapies, acute and extensive cases usually require further treatment modalities [4].

Minimally invasive procedures using advanced endovascular techniques are available with a short treatment time, potentially salvaging a limb-threatening or even life-threatening condition [5]. Examples of this are catheter-directed thrombolysis, pharmacomechanical catheter-directed thrombolysis, aspiration mechanical thrombectomy (AMT), stenting, percutaneous transluminal angioplasty (PTA), and inferior vena cava (IVC) filters placement as a treatment and complication prevention for DVT [6,7]. In patients with increased bleeding risk, AMT is a novel promising therapy with minimal bleeding complications and allows definitive treatment for acute DVT [8]. On the other hand, open surgical thrombectomy assists in removing a more distal DVT or in whom thrombolytic therapy is contraindicated [9]. Furthermore, early thrombus removal has several potential benefits including preservation of valvular patency [10], prevention of post-thrombotic syndrome (PTS) [11], or even in a more life-threatening condition such as phlegmasia cerulea dolens [12,13].

Currently, there is yet to be a consensus regarding the technical combination of endovascular treatments for DVT. In this case report, we presented a patient with acute-on-chronic massive DVT successfully treated with a combination of IVC filter, AMT, stent placement, PTA, and open surgical thrombectomy.

## Case illustration

A 47-year-old woman presented to the emergency department, complaining of significant swelling on her left leg with severe pain (numeric rating scale of 9). Her complaints intensified 4 hours prior to entering the hospital. The patient also had a recurring fever in the past 4 hours. One week earlier, the patient had gone through an uneventful robotic hysterectomy for uterine fibroids. Physical examination showed a red-bluish left leg with pitting edema, followed by laboratory results showing elevated levels of C-reactive protein (53.81 mg/dL), fibrinogen (491 mg/dL), and D-dimer (761 ng/mL). Subcutaneous enoxaparin, 6000 anti-XaIU/0.6 mL 2 times a day (Lovenox, NJ) was administered as an initial anticoagulation therapy.

**Fig. 1:**
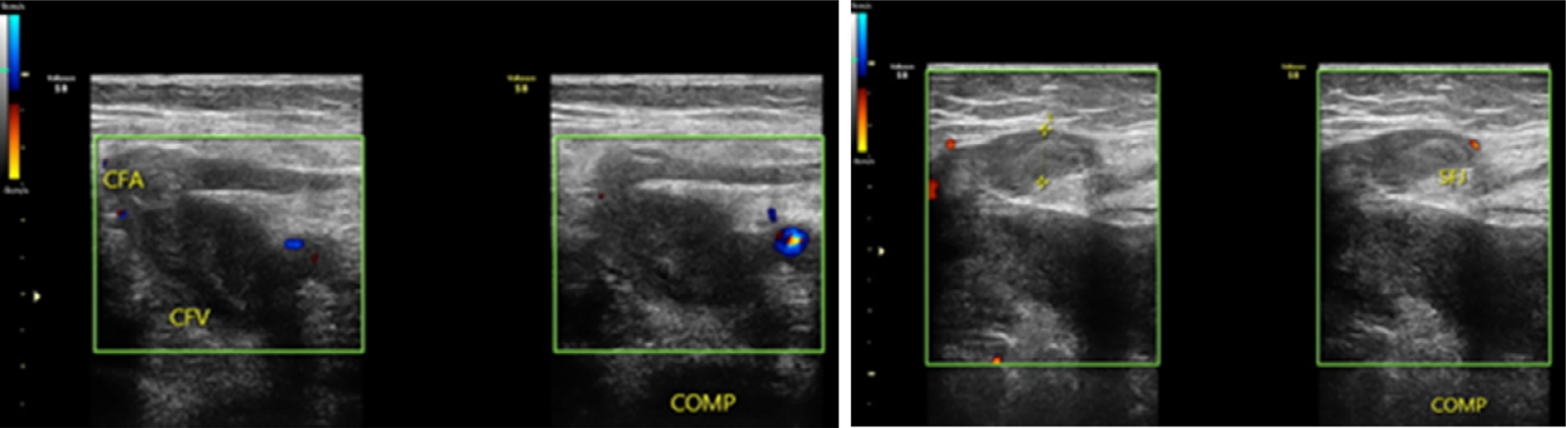
Left lower extremity Doppler ultrasound examination shows no flow in a noncollapsible vein with thrombus in the lumen of the left greater saphenous vein in section with the left common femoral vein (left) and left superficial femoral vein (right).

**Fig. 2:**
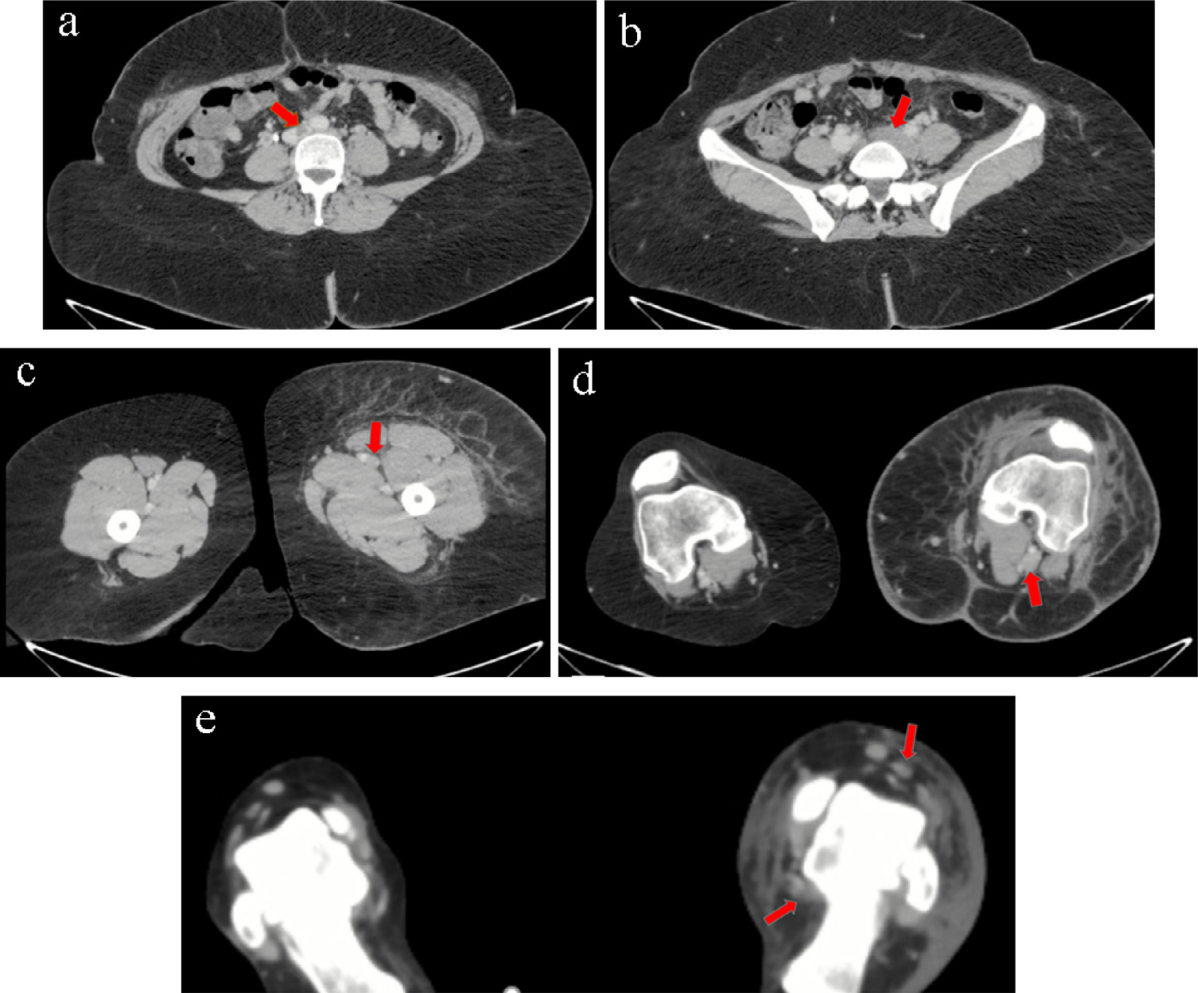
Lower extremities CT angiography shows no flow with extensive thrombus fills the lumen of the distal IVC (A), left common iliac vein (B), left superficial femoral vein (C), left popliteal vein (D), left distal posterior tibial vein and distal anterior tibial vein (E). Also note that there is no contrast filled the lumen of the vein, indicating total occlusion. IVC, inferior vena cava.

Examination by Doppler ultrasound displayed a thrombus on the left greater saphenous vein, left common iliac vein, until the superficial femoral vein. With the compression test, the veins did not collapse upon compression (Fig. 1). Further CT angiography examination showed extensive thrombus from the distal IVC, left common iliac vein, superficial femoral vein, popliteal vein until the anterior tibial vein, posterior tibial vein, and dorsal pedis vein. Prominent soft tissue swelling from the left leg to the left foot was also seen, with the left leg diameter approximately 1.5 times wider than the right leg (Fig. 2). Due to the vast extent of the thrombus, we planned a multimodal management by a collaboration of interventional radiologists and cardiovascular thoracic surgeons.

**Fig. 3:**
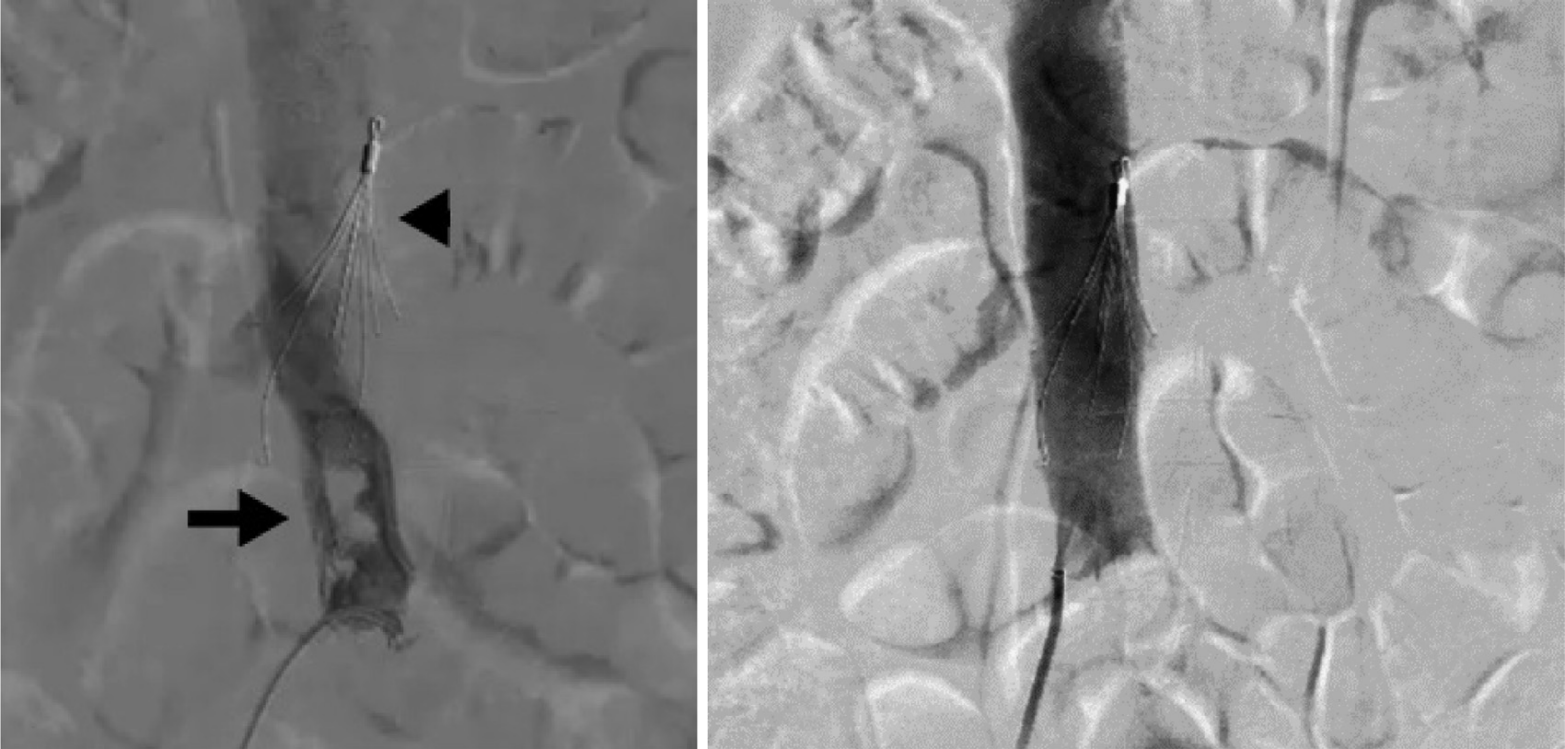
Venogram before and after successful mechanical aspiration thrombectomy. Left: visualization of thrombus (arrow) in the distal IVC with prior IVC filter placement (arrowhead). Right: no visualization of residual thrombus after aspiration thrombectomy in the distal IVC. IVC, inferior vena cava.

The patient underwent an endovascular approach with initial vascular access in the right femoral vein. Venographic examination with the RHA Revass Hydrophilic Angiographic Catheter (APTMedical Inc, Shenzhen, China) confirmed the thrombus in the distal IVC. First, the IVC Filter (ALN Vena Cava Filter, Aln Implants Chirurgicaux, Chemin du Niel, Bormes les Mimosas, France) was placed as a mechanical prophylaxis of pulmonary embolism (PE). Then, the AMT procedure was conducted in the IVC using the Indigo CAT8 Mechanical Thrombectomy Catheter and Indigo Separator 8 as parts of Penumbra's Indigo Aspiration System (Penumbra Inc, Alameda, CA), leaving no residual thrombus (Fig. 3).

**Fig. 4:**
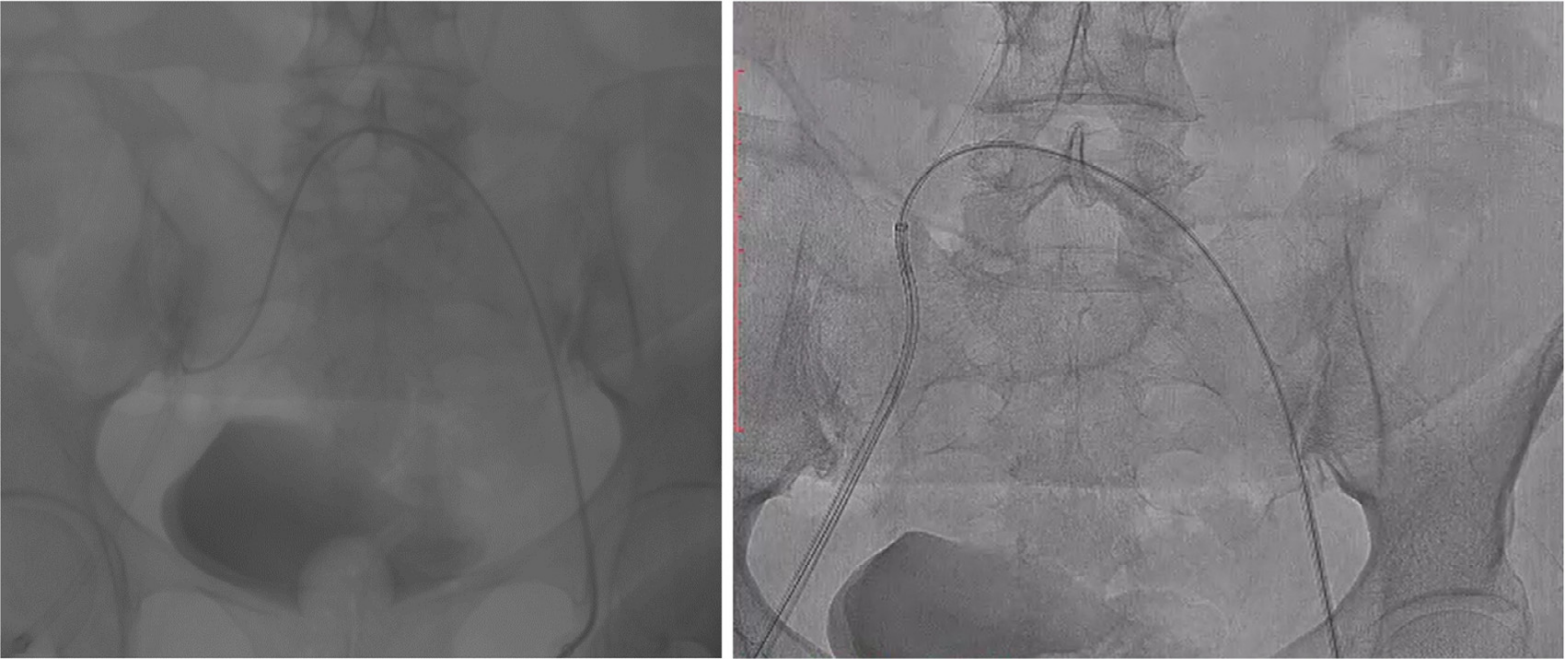
Kissing catheter technique. Left: before the kissing technique. Right: after the kissing and guidewire insertion to the opposite (right leg) introducer sheath.

After the IVC was cleared, the thrombus in the left common iliac vein was too consolidated and could not be penetrated by the wire and the catheter from the initial (right leg) puncture site. For this matter, new access with ultrasound-guided was made in the left common femoral vein. The inserted guidewire and diagnostic catheter managed to pass through the thrombus in the left external iliac vein and the left common iliac vein. With the help of the diagnostic catheter, the guidewire from the left access was crossed to the right side, through the right introducer sheath, until it came out from the patient's right vascular access. The diagnostic catheter from the left leg was pushed until it “kissed” (could be called as “kissing catheter” technique) with the tip of the right introducer sheath, inserted, and fitted tightly inside the introducer sheath (Fig. 4).

The previous guidewire from the left side was exchanged with a more solid, angled type Technowood 0.035” SSS Angiographic Guidewire (Technowood Corporation, Adachi, Tokyo, Japan) from the right side. This allowed us to mount with the same size of the aspiration thrombectomy set, while preparing for further PTA or adjunctive stent if needed. Due to the impenetrable thrombus in the left proximal superficial femoral vein, the guidewire was only able to reach the right common femoral access.

**Fig. 5:**
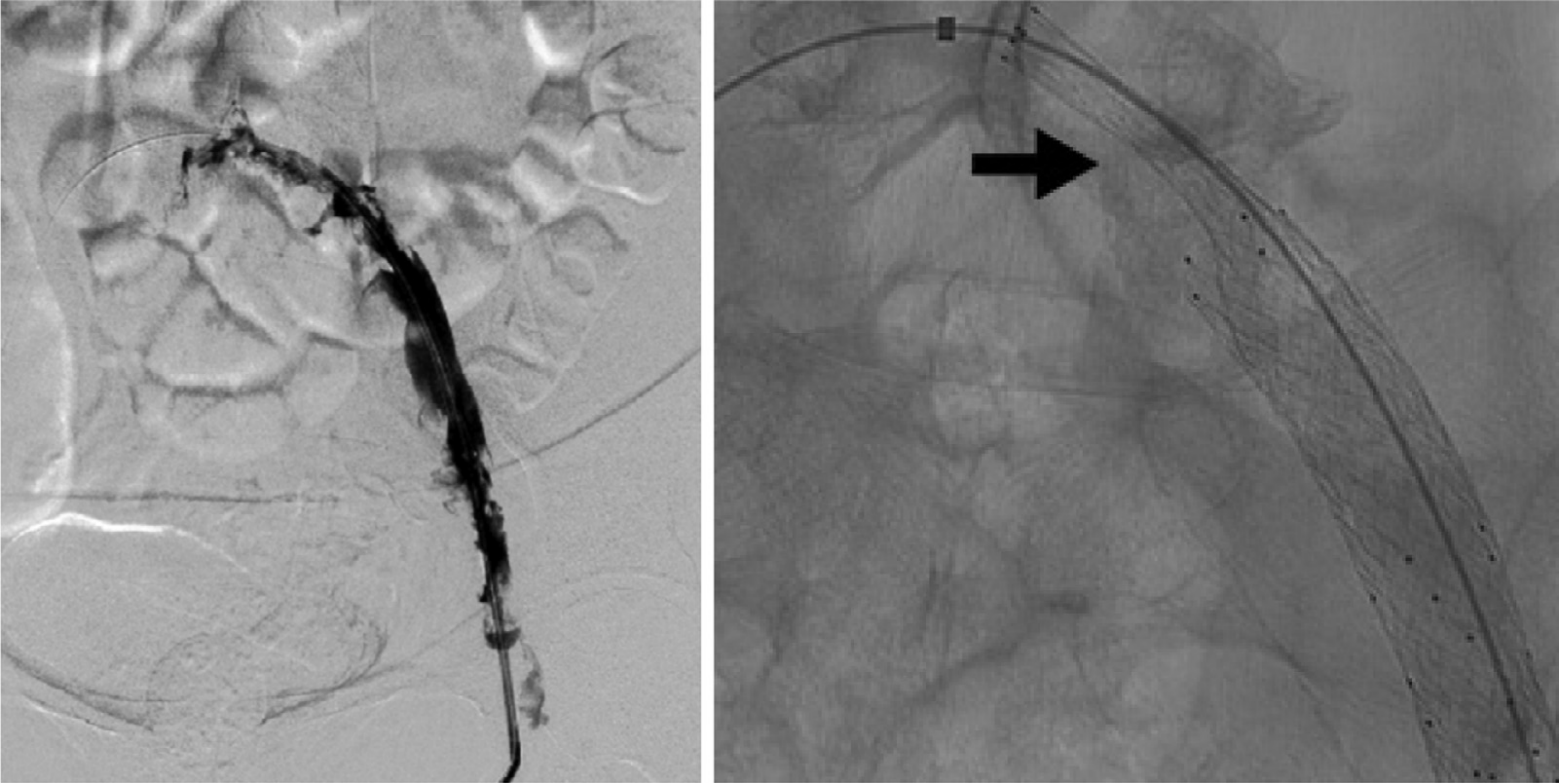
Left: aspiration thrombectomy partially opened the left external iliac vein while the left common iliac vein remains stenotic. Right: stent placement from the left femoral vein to the left common iliac vein, with the stent not fully opened in the proximal site of the left common iliac vein (arrow).

**Fig. 6:**
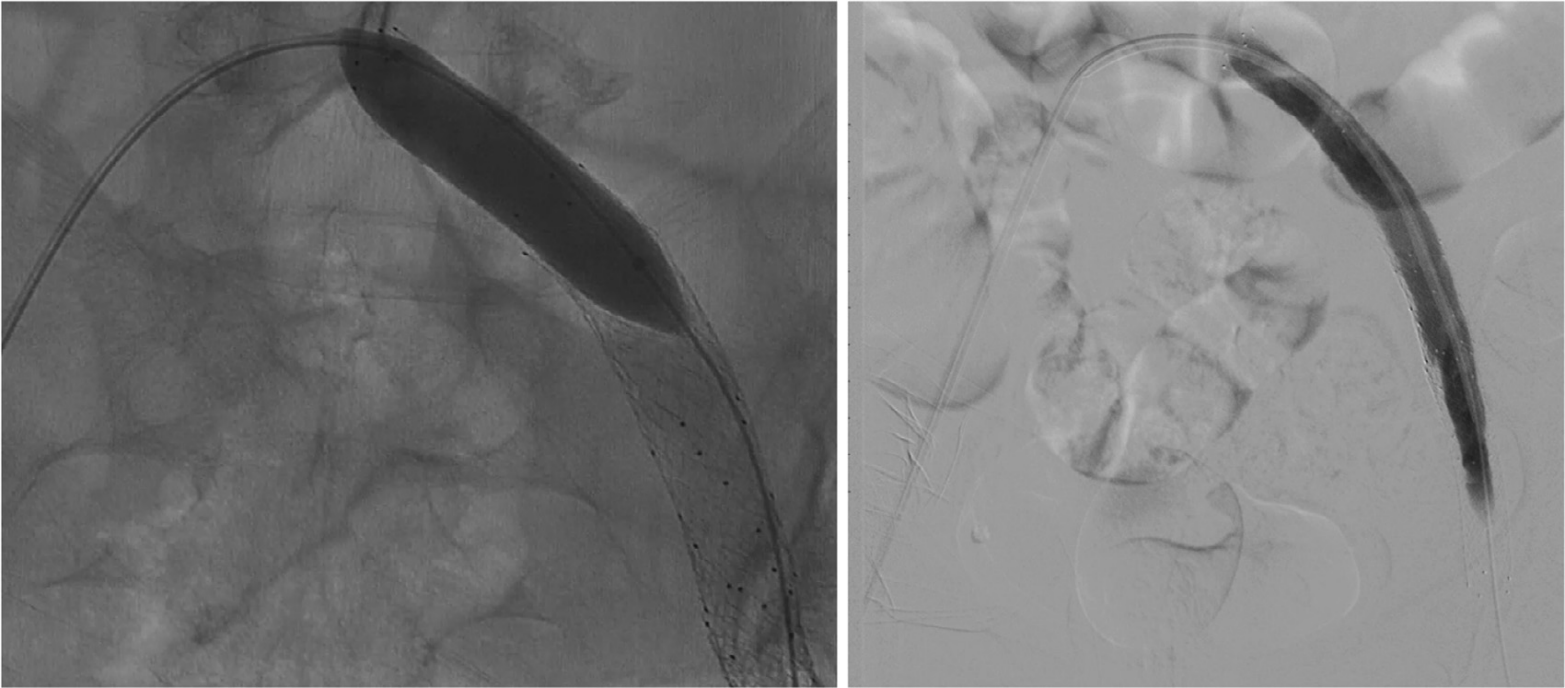
Left: PTA/ballooning of the left common iliac vein. Right: venographic examination after stent placement and PTA showing contrast-filled left femoral vein and left common iliac vein without any thrombus. PTA, percutaneous transluminal angioplasty.

Afterward, aspiration thrombectomy was inserted through the right access and successfully opened most of the external femoral vein until the left distal common iliac vein. However, the proximal left common iliac vein remained stenotic due to the chronic thrombus (Fig. 5) suggestive of a more acute-on-chronic type DVT. To deal with the stenotic left proximal common iliac vein, we proceed to the stenting procedure by using the Epic Self-Expanding Nitinol Stent with Delivery System, 12 mm x 40 mm and 10 mm × 60 mm (Boston Scientific, Galway, Ireland), from the left common femoral vein until the stenotic left proximal common iliac vein, but the stent still failed to fully open the proximal left common iliac vein (Fig. 5). Further management with PTA (or ballooning) using the Mustang PTA Balloon Dilation Catheter, 6F/8.0 mm × 60 mm and 7F/14 mm × 60 mm (Boston Scientific, Galway, Ireland) was performed (Fig. 6). Venographic examination showed a fully dilated proximal common iliac vein without any residual thrombus visualization from the distal veins until the IVC (Fig. 6).

**Fig. 7:**
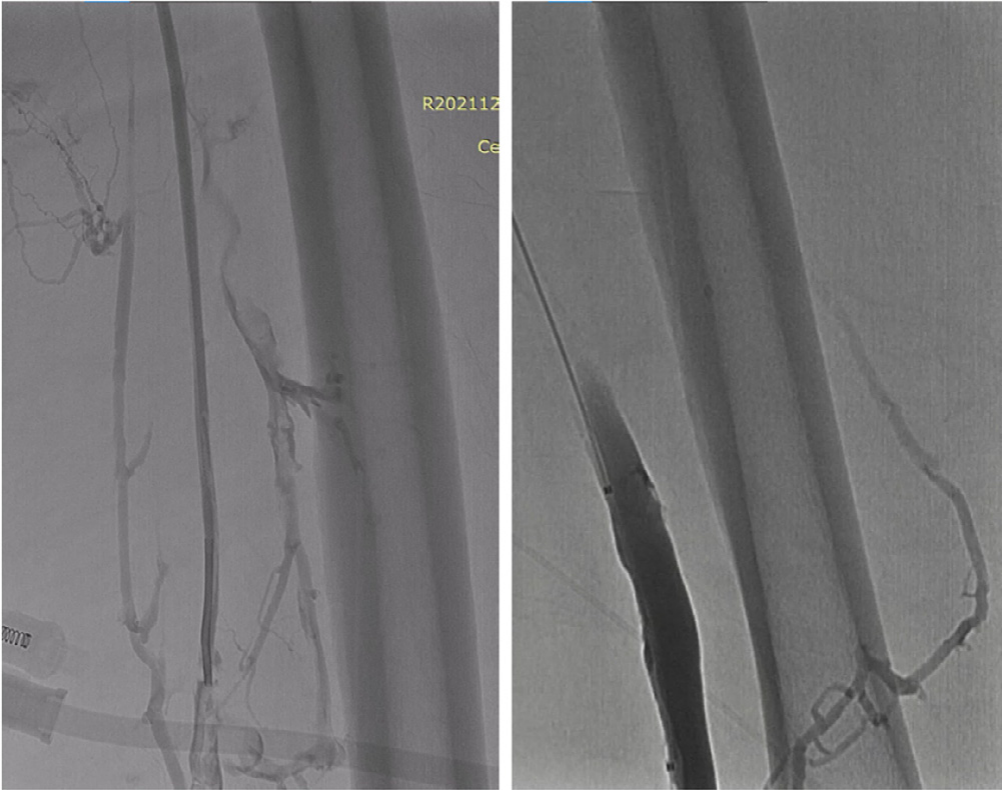
Venogram showing fully contrast-filled lumen after open surgical thrombectomy in the left great saphenous vein (left) and left superficial femoral vein (right).

Open surgical thrombectomy was conducted using the 6F Fogarty Venous Thrombectomy Catheter (Edward Lifesciences LLC, Irvine, CA), which was inserted through the left common femoral access. The thrombus in the distal branches of the left saphenous vein and the left superficial femoral vein were successfully removed (Fig. 7). The thrombus from the popliteal vein to the pedal vein was not removed through thrombectomy due to the need to open the popliteal vein for better reach, with the risk of flow disruption of the more proximal site. The thrombus was expected to dissolve through the postoperative anticoagulation management.

**Fig. 8:**
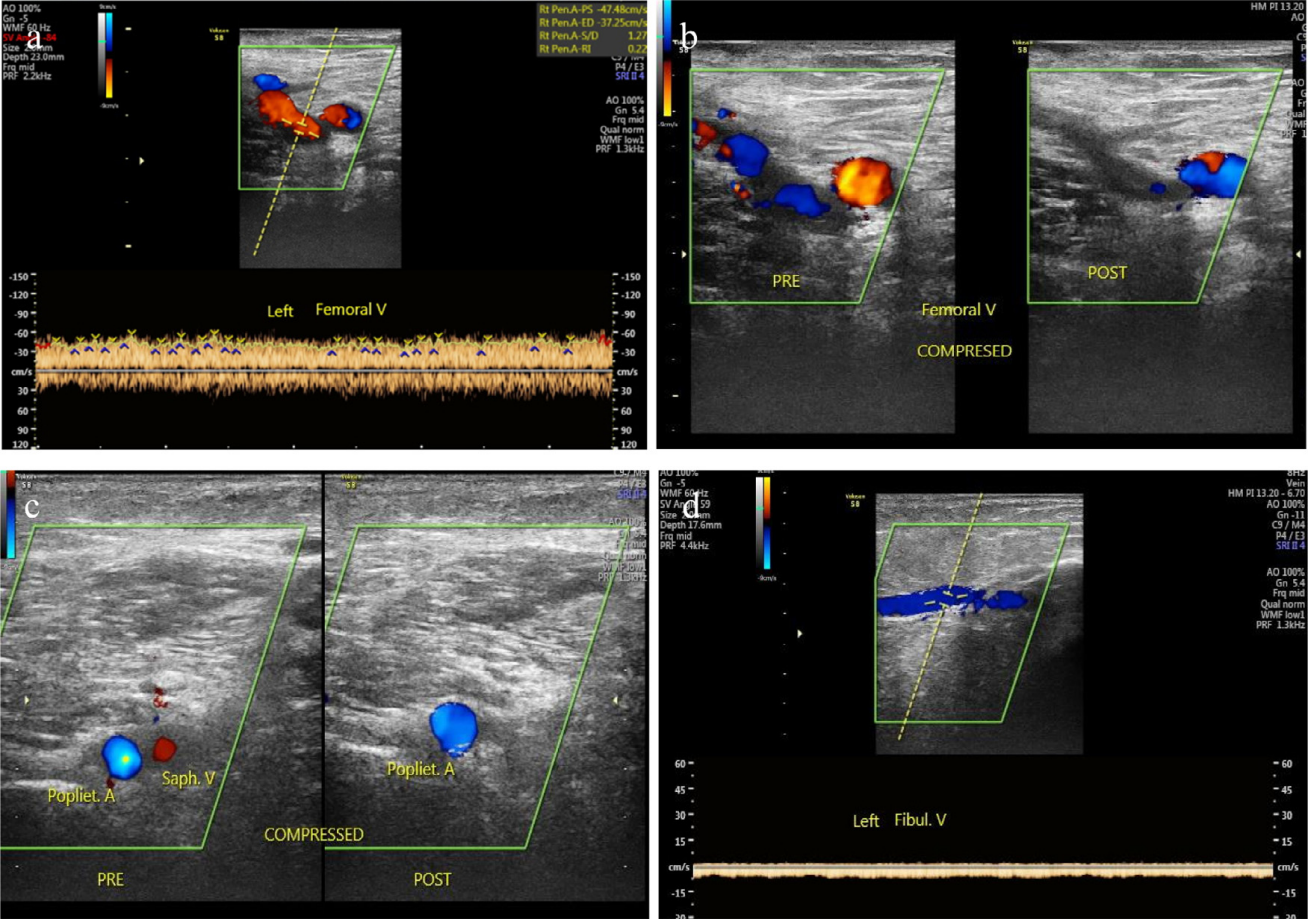
Doppler ultrasound on the left femoral vein (A), compressible left femoral vein (B), left popliteal vein (C), and left fibular vein (D) without any thrombus or stenosis 3 months after the treatment.

After the procedure, the patient was observed in the hospital for 2 more days, followed by outpatient management continuing the enoxaparin and the leg stocking. The patient was routinely evaluated for 3 months and there was no clinical sign or symptoms of recurrence or complication. At the end of the follow-up period, we did a color Doppler ultrasound examination (Fig. 8), showing no thrombus from the iliac vein to the pedal vein and without any rethrombosis or stenosis.

## Discussion

This case successfully demonstrated multimodal management of an acute-on-chronic massive DVT with satisfactory results. The patient had just undergone an operation (hysterectomy) for uterus malignancy and experienced immobilization due to hospitalization, which are known risk factors for DVT. Virchow's Triad (hypercoagulable state, altered blood flow, and endothelial dysfunction) is a long-known pathophysiology for venous thromboembolic events, including DVT [14]. The pathomechanism of the cancer-related venous thromboembolic event is mainly due to both direct and indirect activation of platelets and several coagulation cascades, leading to a hypercoagulable state [15]. On the other hand, immobilization contributes to the stasis of blood flow [16].

Our patient was presented with a possibility of more beneficial results from advanced invasive treatment instead of a conservative pharmacologic approach due to the acute and extensive presentation. Although generally there was limited evidence regarding mechanical thrombus removal to become a first-line approach [3,17], previous studies are showing promises of mechanical thrombectomy efficacy to reduce bleeding and hemolysis complication. A previous retrospective study by Lopez et al. [8] reviewed the use of AMT as a treatment for acute iliofemoral or central DVT, also using the Penumbra's Indigo Aspiration System, in 10 patients. The result was a technical success (defined as resolution of >70% clot without the need of adjunctive thrombolysis) of 60% for AMT as a definitive treatment [8]. A systematic review by Wong et al. [18] also showed a venous patency rate of 75%-100% in acute iliofemoral DVT receiving percutaneous mechanical thrombectomy. Another prospective multicenter study (PEARL registry) for the use of endovascular thrombectomy for lower extremity DVT showed a successful clot removal under 24 hours of procedure time in 73% patient with or without the help of catheter-directed thrombolysis, with 83% of them did not experience rethrombosis after 12-months follow-up [7].

Our patient had an extensive thrombus, including the thrombus in the IVC, distal deep veins involvement, and a persistent thrombus in the proximal common iliac vein. Due to this matter, we approached the patient with a multimodality treatment including IVC filter placement for PE prevention, open surgical thrombectomy for the distal thrombus, and both stent and PTA for the persistent thrombus. Open surgical thrombectomy is indicated for a more distal DVT and in patient contraindicated for thrombolytic therapy [9]. Although seemingly invasive, open surgical thrombectomy providing rapid decompression and good clinical outcomes without increasing the risk of complications [19,20]. Although a routine use of IVC filter is not recommended in DVT, it might be beneficial and should be considered in patients with a thrombus extending to the IVC [9]. The main reason for IVC filter placement is to prevent the occurrence or the recurrence of PE, thus is recommended in patients with high risk of PE development, including a thrombus in IVC and in patient receiving thrombectomy [21]. Adjunctive stent placement and PTA are also beneficial with the consideration of known anatomic location that contributed to the DVT or increased the recurrence risk [22]. Stent placement is preferred in the iliac veins and might extend to the IVC and common femoral vein, while stenting in the femoropopliteal segment showed poor results [9].

There are several risk factors that could lead to DVT complications and recurrences. PTS, a long-term complication consisting of chronic venous symptoms with or without signs secondary to DVT, occurred in about 30%-50% within 2 years after proximal DVT and 5%-10% of them were severe [3]. Several known assessment scores for recurrence risk comprising Vienna prediction model [23], DASH score [24], and HERDOO-2 [25] were including post coagulation therapy D-dimer level, male sex, proximal location, old age, obesity, and history of PTS as the risk factors for recurrent VTE. When compared with catheter-directed thrombolysis, percutaneous AMT was also shown to reduce PTS and bleeding complications in the treatment of acute iliofemoral DVT [18]. Previous meta-analysis by Casey et al. [26] showed a reduction in the risk of PTS and venous obstruction in patient receiving surgical thrombectomy compared to systemic anticoagulation. Until the end of the follow-up period, our patients did not experience any complication or recurrent DVT, although proximal location and obesity as recurrence risk factors were present.

Besides the successful management of our patient, several aspects of our study should be noted. Due to the limited evidence, both aspiration thrombectomy and surgical thrombectomy are not the first-line choices for DVT [3,4,10]. However, the multimodal management in our case, supported by the current evidence, showed the potential of both endovascular and open surgical approach in alleviating symptomatic DVT without any complication. Multimodal treatment might not be needed in a milder DVT case that can be successfully treated with less therapy. The expensive treatment costs, the facility level, and the availability of trained operators were also to be considered.

## Conclusion

Our case demonstrates successful multimodal management through mechanical aspiration thrombectomy, open surgical thrombectomy (Fogarty catheter), PTA, and stenting as a technical approach for acute-on-chronic massive DVT. Despite the limited evidence of AMT and open surgery thrombectomy, previous studies show promising efficacy while reducing the risk of complications and recurrence. Although our research emphasizes the consideration of both percutaneous and surgical approaches in DVT, further studies are needed to increase the evidence of invasive approaches compared to pharmacologic or pharmacomechanical approaches.

## Patient consent

The authors have obtained consent from the patient for their data, including their medical history and imaging studies, to be published in this case report.
